# Inhaled Phosphodiesterase 4 (PDE4) Inhibitors for Inflammatory Respiratory Diseases

**DOI:** 10.3389/fphar.2020.00259

**Published:** 2020-03-12

**Authors:** Jonathan E. Phillips

**Affiliations:** Department of Inflammation Research, Amgen Research, Thousand Oaks, CA, United States

**Keywords:** asthma, COPD, inhalation, CHF6001, pulmonary

## Abstract

PDE4 inhibitors can suppress a variety of inflammatory cell functions that contribute to their anti-inflammatory actions in respiratory diseases like chronic obstructive pulmonary disease (COPD) and asthma. The systemically delivered PDE4 inhibitor roflumilast has been approved for use in a subset of patients with severe COPD with chronic bronchitis and a history of exacerbations. Use of systemically delivered PDE4 inhibitors has been limited by systemic side effects. Inhaled PDE4 inhibitors have been considered as a viable alternative to increase tolerability and determine the maximum therapeutic potential of PDE4 inhibition in respiratory diseases.

## Introduction

Phosphodiesterase (PDE) enzymes metabolize the intracellular second messengers cyclic adenosine monophosphate (cAMP) and cyclic guanosine monophosphate (cGMP). The PDE superfamily of enzymes contains 11 gene families (PDE1 to PDE11), most of which contain several PDE genes (Baillie et al., 2019). PDE4 enzymes are a family of four genes (PDE4A-D) within the PDE superfamily which specifically hydrolyze the 3′,5′ phosphodiester bond of cAMP to yield 5′adenosine monophosphate (5′-AMP). PDE inhibitors have been intensely investigated for airway inflammation due to the clinical efficacy of the non-selective PDE inhibitor theophylline (Krzanowski and Polson, 1988). The expression of PDE4 has been demonstrated in many of the inflammatory cells (T-cells, eosinophils, neutrophils, monocytes, and others) relevant in asthma and COPD (Page, 2014). Therefor PDE4 inhibitors are an effective therapeutic strategy for inflammatory respiratory diseases as they inhibit the hydrolysis of cAMP (Rall and Sutherland, 1958), effectively increasing levels of cAMP, and activating downstream phosphorylation cascades (Li et al., 2018) which relax airway smooth muscle and inhibit inflammation (Houslay et al., 2005; Mulhall et al., 2015).

Three PDE4 inhibitor drugs are currently approved for the treatment of skin or lung diseases: apremilast, crisaborole, and roflumilast. Roflumilast is currently the only PDE4 inhibitor approved for the treatment of a subset of patients with severe COPD. In large clinical trials, roflumilast significantly improved lung function and reduced the rate of exacerbations in patients with severe COPD (Calverley et al., 2009), especially when added to long acting bronchodilators (Fabbri et al., 2009). In a COPD ‘chronic bronchitis’ responder group, namely those suffering from severe airflow obstruction with symptoms of chronic cough and sputum and a history of previous exacerbations, Roflumilast was approved by the FDA in 2011 despite its relatively poor tolerability (Cazzola et al., 2016). Roflumilast has also been studied in asthma and while it has no effect on the acute phase response (bronchoconstriction), it attenuates the late phase asthmatic response and prevents the subsequent increase in bronchial reactivity following an allergen challenge (van Schalkwyk et al., 2005; Louw et al., 2007). Although PDE4 is present in airway smooth muscle cells, selective PDE4 inhibitors have not demonstrated acute bronchodilator effects in humans (Boswell-Smith et al., 2006). The change in FEV_1_ seen in the late phase response is thus mostly due to the resolution of underlying airway inflammation. The primary problem with oral PDE4 inhibitors is the low therapeutic index of these compounds, which severely limits the dose that can be given. Mechanism based adverse effects include nausea, emesis, diarrhea, and headache. It is likely that the maximum tolerated dose of roflumilast is near the bottom of the efficacy dose-response curve.

A potential approach to improve the therapeutic index of orally bioavailable PDE4 inhibitors is to direct the PDE4 subtype selectivity toward PDE4B which accounts for many of the anti-inflammatory effects (Ariga et al., 2004) and away from PDE4D which is related to emesis (Robichaud et al., 2002). It has been demonstrated that a non-subtype selective PDE4 inhibitor like roflumilast has a better therapeutic index that a PDE4D selective inhibitor like cilomilast (Baillie et al., 2019). Unfortunately, no selective PDE4B inhibitors have advanced to clinical trials (Fox et al., 2014). Selectively targeting the low-affinity rolipram-binding site conformer of PDE4 over the high-affinity rolipram-binding conformer (LARBS/HARBS) has also been suggested to reduce the side effects of PDE4 inhibitors (Moretto et al., 2015).

Inhaled administration represents another potential approach to improve the therapeutic index of PDE4 inhibitors. Many PDE4 inhibitors have been designed for inhaled administration in respiratory diseases. A few of these inhaled PDE4 inhibitors have advanced into clinical trials for treatment of asthma and COPD. The inhaled PDE4 inhibitors have been previously reviewed (Yeadon et al., 2010; Tenor et al., 2011; Matera et al., 2014; Mulhall et al., 2015; Spina and Page, 2017). This review is a brief update summarizing the chemical structure, pharmacological, and clinical details of inhaled PDE4 inhibitors.

## Inhaled PDE4 Inhibitors

**FIGURE 1:**
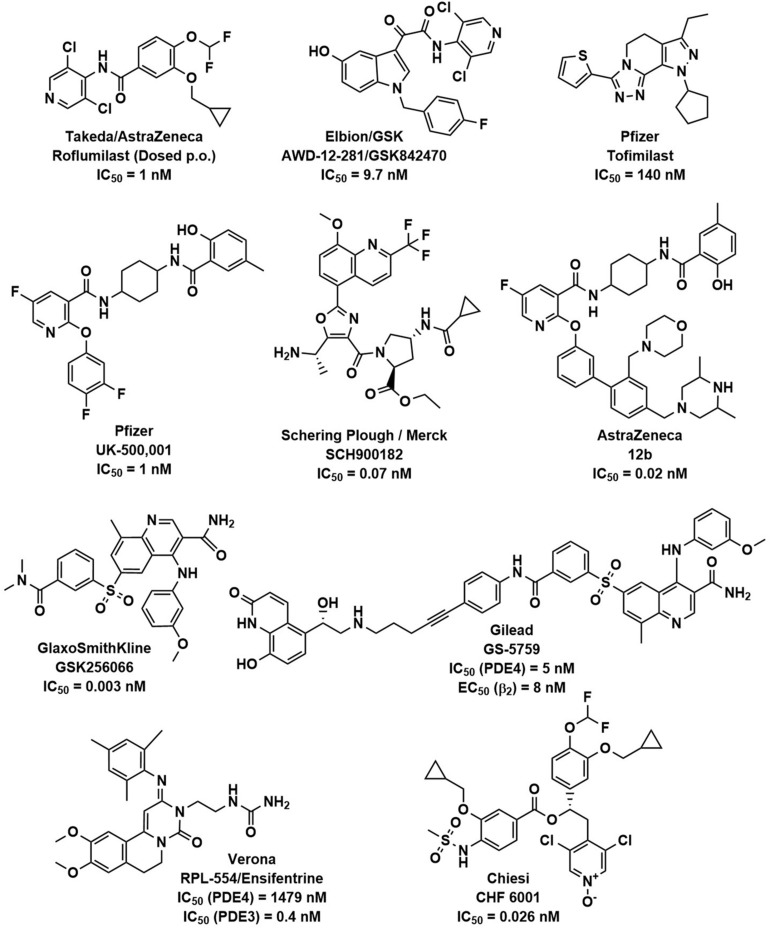
PDE4 inhibitors. The chemical structures of roflumilast and a representative set of inhaled PDE4 inhibitors are shown with the companies that have developed them, as well as their potency (IC_50_) to inhibit the PDE4 enzyme.

Several companies have disclosed data on inhaled PDE4 inhibitors. Figure 1 shows the structures of these compounds and the cell free enzyme-based potencies. Direct comparison between molecules is complicated as data was generated in different labs, using different PDE4 enzymes and experimental protocols.

### AWD-12-281, Elbion/GSK

AWD-12-281 was the first moderately potent PDE4 inhibitor (IC_50_ = 9.7 nM) designed for inhaled delivery (Draheim et al., 2004; Gutke et al., 2005) and showed preclinical efficacy in a variety of species (Kuss et al., 2003). It was in clinical development for asthma and COPD although results are unavailable in the public domain. Development was discontinued due to poor efficacy in 2006.

### Tofimilast, Pfizer

Tofimilast is the least potent (IC_50_ = 140 nM) of the inhaled PDE4 inhibitors reviewed (Duplantier et al., 2007). Clinical trials in mild asthma (Fregonese et al., 2007), persistent asthma (Danto et al., 2007b), GOLD stage II and III COPD patients (Danto et al., 2007a), and LPS challenged healthy subjects (Tan et al., 2007) failed to demonstrate efficacy at any dose and development has been discontinued.

### UK-500,001, Pfizer

UK-500,001 is a moderately potent PDE4 inhibitor (IC_50_ = 1 nM) that showed preclinical efficacy in a variety of species (Vestbo et al., 2009), however, a clinical trial in moderate to severe COPD patients failed to demonstrate efficacy at any dose (Vestbo et al., 2009). A higher incidence of the systemic PDE4 inhibitor-related side effect of diarrhea was observed in the clinical trial despite the lack of efficacy in the lung. Interestingly, the compound was designed to have high plasma protein binding and metabolic clearance to minimize systemic exposure. It has been hypothesized that the lack of clinical activity could be due to its moderate potency and low solubility (De Savi et al., 2014). UK-500,001 was discontinued in 2006 due to lack of efficacy in COPD patients (Yeadon et al., 2010) and the results of the clinical study raised doubt about the potential of inhaled PDE4 inhibitors in COPD.

### GSK256066, GlaxoSmithKline

GSK256066 is the most potent (IC_50_ = 0.003 nM) of the PDE4 inhibitors reviewed and has anti-inflammatory effects in preclinical animal models of pulmonary inflammation (Nials et al., 2011; Tralau-Stewart et al., 2011). Clinical trial data for GSK256066 in mild asthmatics demonstrated significantly reduced early and late asthmatic responses to allergen challenge (Singh et al., 2010), however, in mild COPD patients, no statistically significant changes in inflammatory markers were detected (Watz et al., 2013). It has been hypothesized that the concentration of free compound in the lung was too low to exert a pharmacological effect due to the low solubility and lipophilic nature of the compound (De Savi et al., 2014). Also, potentially hampering clinical development, a low therapeutic index for GSK256066 was revealed by a 14-day inhalation toxicology study in rats (Moretto et al., 2015). GSK256006 was no longer listed on the GSK development pipeline in 2012.

### SCH900182, Schering Plough/Merck

SCH900182 is a highly potent PDE4 inhibitor (IC_50_ = 0.07 nM) that showed preclinical efficacy in a variety of species (Chapman et al., 2010a, b; Ting et al., 2013), yet the compound has not advanced past preclinical development.

### 12b, AstraZeneca

AstraZeneca designed a highly potent phenoxypyridinylamide inhaled PDE4 inhibitor 12b (IC_50_ = 0.02 nM) as a structurally differentiated back-up series to their earlier pyridopyrimidinedione series (De Savi et al., 2014). 12b has preclinical anti-inflammatory activity in the lung with high plasma protein binding and low bioavailability to reduce systemic side effects (De Savi et al., 2014). It is unclear if this compound is progressing into clinical trials as AstraZeneca currently has no PDE4 inhibitors in their pipeline^1^.

### GS-5759, Gilead

GS-5759 is a bifunctional, moderately potent PDE4 inhibitor (IC_50_ = 5 nM) and β_2_ adrenergic agonist (EC_50_ = 8 nM) made by linking a GSK256066 structurally related PDE4 inhibitor to an indacaterol structurally related β_2_-agonist (Tannheimer et al., 2014; Joshi et al., 2017). Although it has shown preclinical efficacy in a variety of species (Salmon et al., 2014) this program is assumed to be discontinued as no development has been reported.

### Almirall

Almirall most recently published on two potent chemical series, the naphthyridinones (represented by compound 72, IC_50_ = 0.17 nM) (Roberts et al., 2018) and pyridazinones (represented by compound 50, IC_50_ = 0.05 nM) (Gracia et al., 2016). The compounds are not planned to advance past preclinical development.

### Lotamilast, Eisai

Lotamilast is a moderately potent PDE4 inhibitor (IC_50_ = 2.8 nM) that effectively suppresses LPS induced neutrophilic pulmonary inflammation when delivered to mice by dry powder insufflation (Kubota et al., 2015). Eisai licensed the drug to Roivant Sciences and lotamilast is currently in clinical development as a topical ointment for Eczema/Atopic Dermatitis.

### Ensifentrine/RPL554, Verona

Ensifentrine is a dual moderately potent PDE3 (IC_50_ = 0.4 nM) and weakly potent PDE4 (IC_50_ = 1479 nM) inhibitor (Boswell-Smith et al., 2006) that has been formulated for dry powder or nebulized delivery. Ensifentrine is included in this review as it is characterized in the literature as dual PDE3 and PDE4 inhibitor, but it is recognized that no reliable evidence is available for its ability to elicit PDE4 inhibitor like anti-inflammatory activity in patients with COPD or asthma (Cazzola et al., 2019) and most of the clinical effects of ensifentrine are likely attributable to PDE3 inhibition alone. PDE3 is the primary isoenzyme in airway smooth muscle where it has effects on airway tone and PDE3 inhibitors have demonstrated acute bronchodilator effects. PDE3 is also found in cardiac and vascular tissue therefor PDE3 inhibitors are likely to have undesirable adverse systemic effects that may be minimized by inhaled delivery. An inhaled dual PDE3/PDE4 inhibitor should have both bronchodilator and anti-inflammatory effects and indeed, in preclinical animal models RPL554 has demonstrated bronchodilator and anti-inflammatory effects (Boswell-Smith et al., 2006). RPL554 has successfully undergone phase 2 studies in both asthma (Bjermer et al., 2019) and COPD (Franciosi et al., 2013; Singh et al., 2018) but has currently only demonstrated significant bronchodilator effects. Verona is planning to begin a final-stage clinical evaluation for nebulized RPL554 treatment of patients with severe COPD in 2020.

### CHF 6001, Chiesi

CHF 6001 is a highly potent PDE4 inhibitor (IC_50_ = 0.026 nM) that showed preclinical efficacy in a variety of species (Moretto et al., 2015; Villetti et al., 2015). It is well tolerated in humans (Mariotti et al., 2018). In a double blind, placebo controlled, three-way crossover study, 36 atopic asthmatics received CHF 6001 doses of 400 or 1200 μg inhaled once daily for 9 days. Allergen challenges were performed on day 9. Both CHF 6001 doses significantly inhibited allergen induced late asthmatic response (Singh et al., 2016). CHF 6001 was well tolerated, with similar numbers of adverse events in each treatment period. In a double-blind, placebo-controlled, three-way crossover study in 61 patients with COPD and chronic bronchitis (already treated with inhaled triple therapy: inhaled corticosteroid, long-acting muscarinic antagonist, and long-acting β_*2*_-agonist) received CHF 6001 doses of 800 or 1600 μg inhaled twice daily for 32 days. Multiple biomarkers of airway inflammation in induced sputum were significantly reduced, demonstrating additional lung anti-inflammatory activity on top of standard of care (Singh et al., 2019). Both doses of CHF 6001 were safe and the incidence of gastrointestinal adverse effects was comparable to placebo. CHF 6001 is currently in phase IIb clinical trials for the treatment of COPD (Stellari et al., 2019). Chiesi also has recently published on two structurally differentiated backup series to CHF 6001 (Armani et al., 2014; Carzaniga et al., 2017). The clinical data for CHF 6001 demonstrates anti-inflammatory effects with minimal systemic side effects, likely due to the high lung relative to systemic exposure provided by inhaled delivery and favorable LARBS/HARBS ratio. The results of the phase III clinical trials are eagerly awaited.

## Discussion

PDE4 inhibitors block the breakdown of cAMP and decrease airway inflammation. Use of PDE4 inhibitors has been limited by mechanism based systemic side effects (nausea, diarrhea, and headache). Inhaled administration of PDE4 inhibitors for the treatment of respiratory diseases applies the drug directly to the site of action, the lungs, and minimizes the systemic exposure. This is an important advantage that can greatly increase the therapeutic index of a drug. Many of the inhaled PDE4 inhibitors highlighted in this review are discontinued (Table 1) and have produced only modest beneficial clinical effects in respiratory disease patients. Only CHF 6001 is currently advancing through clinical development. None of the discontinued inhaled compounds have convincingly covered the target, thus the hypothesis of increased efficacy with avoidance of systemic adverse events was not adequately tested. Since oral roflumilast does demonstrate clinical efficacy, it is possible that a systemic effect of PDE4 inhibitors is required, possibly to suppress cytokine production or cell activity in inflammatory cells before they reach the lung. The recent successful results with CHF 6001, suggest that the systemic biology of PDE4 is less relevant than the lung biology for clinical efficacy in inflammatory respiratory diseases.

**TABLE 1:** Discontinued inhaled PDE4 inhibitors.

Name/Company	Disease indications	Development stage (Last development reported)
AWD-12-281	COPD	Phase II (2006)
Elbion/GSK	Asthma	Phase II (2003)
Tofimilast	COPD	Phase II (2007)
Pfizer	Asthma	Phase II (2007)
UK-500,001	COPD	Phase II (2009)
Pfizer		
GSK256066	COPD	Phase II (2013)
GlaxoSmith Kline	Asthma	Phase II (2010)
12b	COPD	Discovery (2014)
AstraZeneca		
SCH900182	Asthma	Discovery (2013)
Schering Plough/Merck		
GS-5759	COPD	Discovery (2017)
Gilead		

CHF 6001 is currently the only inhaled PDE4 inhibitor advancing through clinical development and already has promising results in phase II clinical trials in asthma (Singh et al., 2016) and COPD (Singh et al., 2019). The clinical data for CHF 6001 demonstrates anti-inflammatory effects while minimizing the typical PDE4 inhibitor induced systemic side effects. This is likely due to the high lung relative to systemic exposure provided by inhaled delivery and demonstrates the therapeutic index of a PDE4 inhibitor can be increased by inhaled delivery.

Inhaled PDE4 inhibitors offer an intriguing new class of treatment for inflammatory respiratory diseases. Oral PDE4 inhibitors like roflumilast only have explored the lower end of the efficacy dose-response curve in man. Assuming toleration issues can be overcome by inhaled delivery, the maximum therapeutic potential in respiratory diseases of PDE4 inhibition will be determined. The pharmacology of inhaled PDE4 inhibitors should minimize side effects driven by biology outside the lung. Their efficacy in asthma and COPD suggest PDE4 inhibitors will reduce inflammation regardless of the level of type 2 inflammation. Their efficacy on top of standard of care in COPD suggest they could be combined with inhaled steroids and bronchodilators. Answers to the questions surrounding efficacy and safety of an inhaled PDE4 inhibitor are eagerly awaited in phase III clinical trials.

## Author Contributions

JP contributed to conception, design, data interpretation, and drafting of manuscript.

## Conflict of Interest

JP is an employee of Amgen Inc., which provides financial support for his work. The funder was not involved in the collection, analysis, interpretation of data, the writing of this article or the decision to submit it for publication.

## Footnotes

1 https://www.astrazeneca.com/our-science/pipeline.html, (accessed December 1, 2019).
